# A multicentre implementation trial of an Artificial Intelligence-driven biomarker to inform Shared decisions for androgen deprivation therapy in men undergoing prostate radiotherapy: the ASTuTE protocol

**DOI:** 10.1186/s12885-025-13622-1

**Published:** 2025-02-13

**Authors:** Eric Wegener, Michael Ng, Mario Guerrieri, Timothy N. Showalter, Jeremy de Leon, Sagar Ramani, Marcus Dreosti, Tee Lim, Bradley Wong, Michael Chao, Kathryn Hogan, Avi Raman, Scott McClintock, Darren Foreman, Matthew Brown, Stephen McCombie, Kevin McMillan, Kieran Beattie, Mark Frydenberg, Lih-Ming Wong, Dickon Hayne, John Yaxley, Phillip Stricker, Jarad Martin

**Affiliations:** 1 GenesisCare Lake Macquarie Private Hospital, 3 Sydney St Lake Macquarie, Gateshead, NSW 2290 Australia; 2 University of Newcastle, Callaghan, NSW 2308 Australia; 3 GenesisCare St Vincent’s Hospital, 41 Victoria Parade, Fitzroy, VIC 3065 Australia; 4 Department of Radiation Oncology, University of Virginia, Charlottesville, Virginia USA; 5 GenesisCare St Vincent’s Hospital, Level A, 438 Victoria Street, Darlinghurst, NSW 2010 Australia; 6 GenesisCare John Flynn Private Hospital, 42 Inland Drive, Tugun, QLD 4224 Australia; 7 Tennyson Centre, GenesisCare Kurralta Park, 520 South Road, Kurralta Park, SA 5037 Australia; 8 GenesisCare Fiona Stanley Hospital, 102–118 Murdoch Drive, Murdoch, WA 6150 Australia; 9 GenesisCare Buderim, 10 King Street, Buderim, QLD 4556 Australia; 10 GenesisCare Ringwood Private Hospital, 36 Mount Dandenong Road, Ringwood East, VIC 3135 Australia; 11 Lingard Specialist Centre, Suite 3/2 Lingard St, Merewether, NSW 2291 Australia; 12 Gold Coast Private Hospital, Level 3, 123 Nerang St, Southport, QLD 4215 Australia; 13 South Terrace Urology, 326 South Tce, Adelaide, SA 5000 Australia; 14 St John of God Wexford Medical Centre, 3 Barry Marshall Parade, Murdoch, 6150 WA Australia; 15 Knox Private Hospital, 262 Mountain Hwy, Wantirna, VIC 3152 Australia; 16 Suite 6, Maitland Specialist Centre, 173 Chisholm Rd, East Maitland, NSW 2323 Australia; 17 Australian Urology Associates, Ground Floor, 322 Glenferrie Rd, Malvern, VIC 3144 Australia; 18 Melbourne Urology Group, Suite 2, 141 Grey Street, East Melbourne, Australia; 19 Medical School, The University of Western Australia, Perth, Australia; 20 Wesley Urology Clinic, Suite 42, Level 4, Wesley Medical Centre, 40 Chasely Street, Auchenflower, QLD 4066 Australia; 21 St Vincent’s Hospital Sydney, Darlinghurst, New South Wales Australia

**Keywords:** (MeSH): prostate cancer, Radiotherapy, Artificial Intelligence, Deep learning, Digital pathology, Biomarkers, Androgen deprivation therapy

## Abstract

**Background:**

Androgen deprivation therapy (ADT) improves outcomes in men undergoing definitive radiotherapy for prostate cancer but carries significant toxicities. Clinical parameters alone are insufficient to accurately identify patients who will derive the most benefit, highlighting the need for improved patient selection tools to minimize unnecessary exposure to ADT’s side effects while ensuring optimal oncological outcomes. The ArteraAI Prostate Test, incorporating a multimodal artificial intelligence (MMAI)-driven digital histopathology-based biomarker, offers prognostic and predictive information to aid in this selection. However, its clinical utility in real-world settings has yet to be measured prospectively.

**Methods:**

This multicentre implementation trial aims to collect real-world data on the use of the previously validated Artera MMAI-driven prognostic and predictive biomarkers in men with intermediate-risk prostate cancer undergoing curative radiotherapy. The prognostic biomarker estimates the 10-year risk of metastasis, while the predictive biomarker determines the likely benefit from short-term ADT (ST-ADT). A total of 800 participants considering ST-ADT in conjunction with curative radiotherapy will be recruited from multiple Australian centers. Eligible patients with intermediate-risk prostate cancer, as defined by the National Comprehensive Cancer Network, will be asked to participate. The primary endpoint is the percentage of patients for whom testing led to a change in the shared ST-ADT recommendation, analyzed using descriptive statistics and McNemar’s test comparing recommendations before and after biomarker testing. Secondary endpoints include the impact on quality of life and 5-year disease control, assessed through linkage with the Prostate Cancer Outcomes Registry. The sample size will be re-evaluated at an interim analysis after 200 patients.

**Discussion:**

ASTuTE will determine the impact of a novel prognostic and predictive biomarker on shared decision-making in the short term, and both quality of life and disease control in the medium term. If the biomarker demonstrates a significant impact on treatment decisions, it could lead to more personalized treatment strategies for men with intermediate-risk prostate cancer, potentially reducing overtreatment and improving quality of life. A potential limitation is the variability in clinical practice across different centers inherent in real-world studies.

**Trial Registration:**

Australian New Zealand Clinical Trials Registry, ACTRN12623000713695p. Registered 5 July 2023.

**Supplementary Information:**

The online version contains supplementary material available at 10.1186/s12885-025-13622-1.

## Background

Prostate cancer is the most commonly diagnosed male malignancy, with over 24,000 cases diagnosed in Australia in 2021 [1]. It is responsible for the highest incidence of cancer-related disability worldwide, with a large proportion of such morbidity because of the adverse outcomes associated with over-treatment or under-treatment [2]. Radiotherapy is commonly employed to cure localised disease, with androgen deprivation therapy (ADT) reserved for treatment intensification of higher stage disease [3]. ADT has toxicities, with the potential to reduce quality of life and cause adverse health outcomes [4]. The challenges for treatment intensification lie in accurate prognostication, with a multitude of tumour, patient, and treatment factors also impacting outcomes.

The National Comprehensive Cancer Network (NCCN) risk classification has helped improve prognostication for localised prostate cancer and remains a widely used tool to guide management [5]. This system is imperfect, with overlapping outcomes between risk groups, as well as a wide range of possible disease control rates within each risk classification, and several alternative systems such as CAPRA have been proposed [6, 7]. Issues of variability and subjectivity can enter into some of the parameters, such as histopathological grading, weakening the prognostic ability. The Gleason scale was developed over half a century ago and has shown ambiguity in reproducibility across expert uropathologists [8]. Decipher, a tissue-based genomic biomarker assessing 22 genes, has shown improved prognostication but lacks validation in prospective randomized trials [9]. There also remain challenges with consumptive pathology tests such as cost, laboratory requirements, processing time, and tumour representation. A deeper issue is that even if a patient with a worse prognosis is identified, whether treatment intensification is likely to benefit that specific individual is unknown. It is therefore important to develop predictive biomarkers to help determine if a specific intervention, such as ADT, will lead to additional efficacy for such an individual.

Several randomized controlled trials (RCTs) have investigated the use of short-term ADT (ST-ADT) in intermediate-risk prostate cancer, including RTOG-9408, EORTC 22,991, DFCI 95–096, and RTOG 0815 [3, 10–13]. While some of these trials have demonstrated improvements in biochemical control and, in some cases, reductions in distant metastases and cancer-specific mortality with the addition of ST-ADT, they often suffer from limitations such as the use of older radiotherapy techniques, heterogeneous patient populations, and an inability to precisely identify the subpopulation most likely to benefit from ADT. The D’Amico RCT highlighted comorbidity as a potential discriminator, suggesting that patients with moderate to severe comorbidities may not benefit from ST-ADT and may experience increased cardiac mortality [11, 12]. These findings underscore the need for better tools to guide personalized treatment decisions regarding ST-ADT in intermediate-risk prostate cancer.

**Fig. 1:**
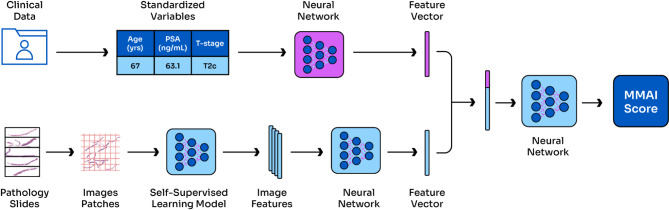
Development of a Multimodal Artificial Intelligence Tool in Prostate Cancer

The ArteraAI Prostate Test uses a multimodal AI (MMAI) architecture that encompasses both clinical and digital histopathology data. Multimodal deep learning uses combinations of various data modalities together, compared to a singular modal learning which would analyse each of these independently. The clinical features of this model consist of: age, PSA, Gleason combined, Gleason primary, Gleason secondary, and T-stage. The second pipeline consists of digital histopathology, which was trained using a self-supervised learning model. This analyzes multiple image features using a neural network. Both the clinical and histopathology vectors are analysed together using a separate neural network to create a MMAI score, Fig. 1.

The ArteraAI Prostate Test is a unique clinicopathological biomarker test which utilises the MMAI architecture described to run two models: a prognostic model and a predictive model. Firstly, the prognostic model provides estimates of distant metastasis (DM) and prostate cancer-specific mortality (PCSM) risk. The prognostic deep learning model was trained and validated on 5 phase III randomised control trials (NRG/RTOG 9202, 9408, 9413, 9910, and 0126), with a total of 5,654 patients and a dataset of 16,204 histopathology slides. This model was shown to significantly outperform the NCCN classification with a 5-year distant metastasis AUC of 0.83 compared to 0.72 for NCCN, *p* < 0.001 [14]. The predictive model assesses the benefit of short-term, 4–6 months of ADT (ST-ADT) in intermediate-risk (IR) prostate cancer patients and has recently been validated. In the predictive model positive patients, ST-ADT significantly reduced the risk of distant metastasis compared to radiotherapy alone (sHR = 0.34, 95% CI 0.19–0.63, *p* < 0.001). There were no significant differences with the addition of ADT in the predictive model negative subgroup (sHR = 0.92, 95% CI 0.59–1.43, *p* = 0.71) [15].

The test can inform the shared ST-ADT discussion between clinicians and patients on the benefit ADT may have in men being managed with definitive RT for IR prostate cancer. The real-world data on the impact of this test in clinical practice is currently lacking, which is the main question we are exploring via ASTuTE.

## Design

The ASTuTE trial (*A*rtificial intelligence *S*teering *T*estosterone deprivation *T*reatments in prostate cancer *E*xternal-beam radiotherapy) is an open-label, multicentre, prospective registry and trial of implementation that aims to collect real world data on the use of a MMAI-driven biomarker digital histopathology test developed by Artera^®^ for use in IR prostate cancer men undergoing curative radiotherapy.

The trial was designed by the authors, using the SPIRIT-AI extension recommendations [16], and was devised to assess the impact of the ArteraAI Prostate Test on shared ST-ADT decisions. The trial was first registered with the Australian Clinical Trials Registry (ACTRN12623000713695) on 5 July 2023. Central ethical approval was obtained from St Vincent’s Human Research Ethics Committee (HREC 2023/ETH01630) in 2023, with the first patient enrolled December 2023. Local ethical and governance approval has been obtained from all participating sites. The study is being conducted in accordance with the Declaration of Helsinki, the National Health and Medical Research Council (NHMRC) National Statement on Ethical Conduct in Human Research 2007 and the NHMRC Australian Code for Responsible Conduct of Research. All participants are providing written informed consent.

**Fig. 2:**
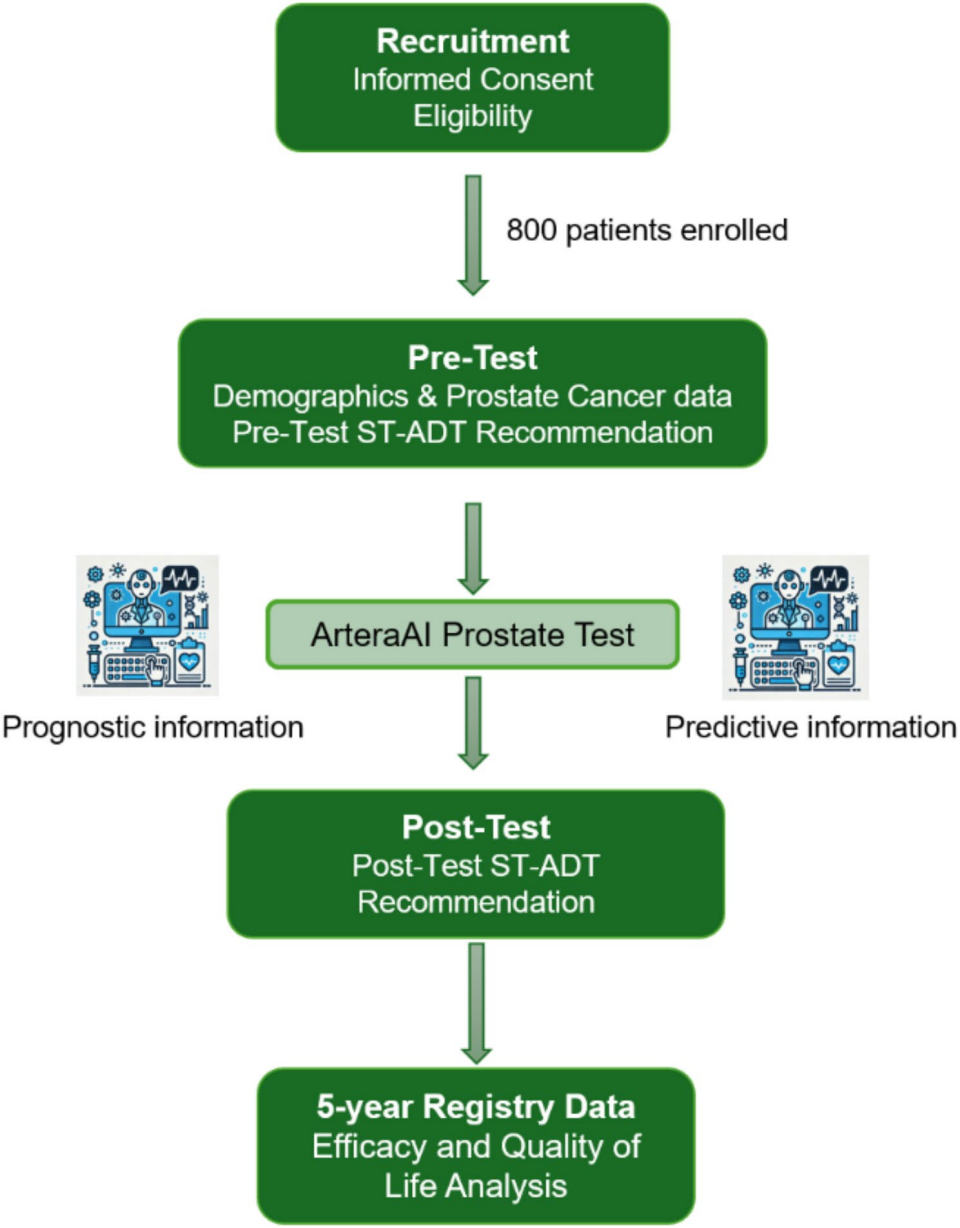
ASTuTE study schema

The primary goal of this study is to create a de-identified database of patients, test results, and treatment decisions that can be queried to determine the clinical utility of the MMAI digital histopathology test known as ArteraAI Prostate Test, in the utilisation of ST-ADT for men with IR prostate cancer. Eligible participants will undergo data collection as per Fig. 2. For patients that will receive radiotherapy, all curative intent radiotherapy dose/fractionation schedules are allowed including conventional [17], moderately hypofractionated [18] and ultra-hypofractioned [19] as well as dominant intraprostatic lesion (DIL) boosting [20]; this will enable data generalizability across modern techniques.

### Endpoints

The primary objective is to assess the impact of the ArteraAI Prostate Test on shared ST-ADT decisions with IR prostate cancer men undergoing curative radiotherapy. To evaluate this, the endpoints of shared ST-ADT decisions pre-test and post-test will be recorded. The primary endpoint is the percentage of cases with changes in ST-ADT shared decision recommendations.

The secondary objectives will be assessed through linkage with the Prostate Cancer Outcomes Registry (PCOR) and will assess efficacy and quality of life. The secondary endpoint of efficacy will be assessed at 5 years using the Phoenix criteria of PSA nadir + 2ng/mL [21], and/or initiation of salvage treatment and/or imaging confirming recurrent disease. The secondary endpoint of quality of life will be assessed using the Expanded Prostate Cancer Index Composite questionnaire (EPIC-26).

The primary hypothesis is that the ArteraAI Prostate Test will result in changes to ADT management of IR prostate cancer patients. The secondary hypotheses are that this will be accompanied by ongoing high rates of disease control and improved quality of life for those spared ADT.

### Eligibility criteria

The target population for the ASTuTE trial is adults with prostate adenocarcinoma and IR per the NCCN risk classification [22]. Potential participants will be screened for eligibility according to the inclusion and exclusion criteria outlined in Table 1.

**Table 1:** Eligibility criteria

Inclusion criteria	Exclusion criteria
1. Adult males > 18years of age	1. Participants who have already commenced ADT or inability to receive ADT.
2. Participants must have intermediate risk, localised adenocarcinoma of the prostate according to NCCN risk Favourable intermediate risk (FIR): • 1 intermediate risk factor (IRF) • Grade Group 1 or 2 (Gleason Score ≤ 6 or Gleason Score 7 {3 + 4}) • < 50% biopsy cores positive (e.g., < 6 of 12 cores) Unfavourable intermediate risk (UIR) • 2 or 3 IRFs • Grade Group 3 (Gleason Score 7) • ≥ 50% biopsy cores positive (e.g., ≥ 6 of 12 cores) IRFs: • Clinical stage cT2b-cT2c • Grade Group 2 or 3 (Gleason Score 3 + 4 = 7 or 4 + 3 = 7) • PSA 10-20ng/mL	2. Participants with insufficient tissue and/or histopathology issues which may arise pertaining to the generation of an accurate ArteraAI Prostate Test result.
	3. Participants with histological or cytological evidence of neuroendocrine or small cell differentiation.
	4. Prostate adenocarcinoma that cannot be International Society of Urological Pathologists (ISUP) graded.
	5. High risk clinical features (PSA > 20, Grade Group 4–5, Stage T3-4). Node positive or presence of distant metastases (cN1 or cM1).
3. Estimated life expectancy > 10 years	
4. Participants must be planned to undergo curative-intent radiotherapy for prostate cancer	
5. Willing and able to provide written informed consent	

### Methods

After providing informed consent, clinical variables including combined Gleason score, Primary Gleason Score, Secondary Gleason Score, clinical T-stage, baseline PSA, and age at biopsy are recorded. Next, a single formalin fixed, paraffin embedded (FFPE) hematoxylin and eosin-stained slide containing one biopsy core with the tumor that has the highest Gleason grade used by the local pathologist in making their diagnosis for the patient will be digitised.

When scanning is completed, a certified pathologist approved by Artera^®^ will review the digitally converted image to assess suitability for the MMAI biomarker test. The test will be run using locked AI models (v1.2) for the duration of the study. The locked models makes sure all participants will be assessed using the same models [23]. After completion of the test, the ArteraAI Prostate Test report will be verified by the certified pathologist and then made available to clinical staff via a web portal.

**Fig. 3:**
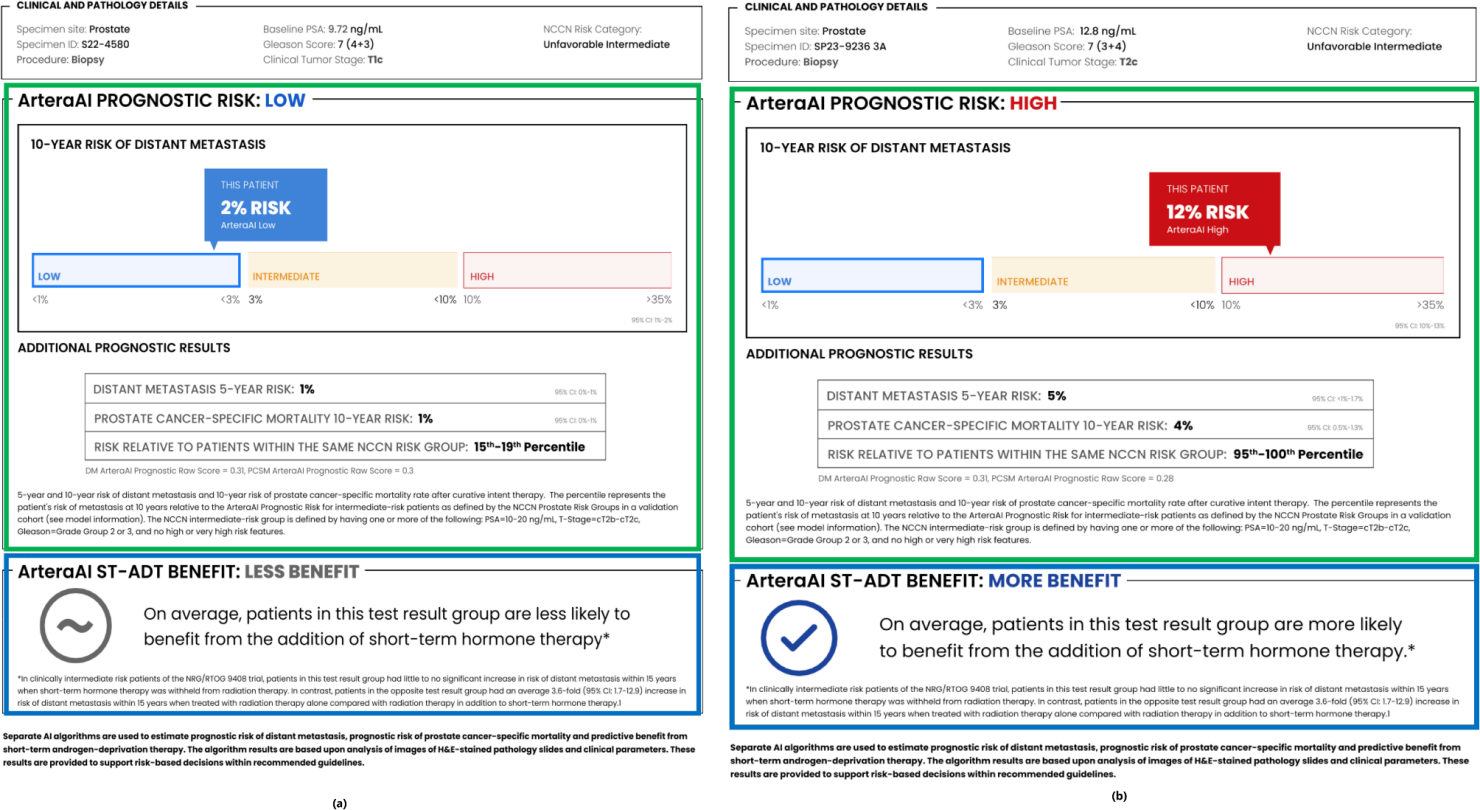
**a and b.** ArteraAI Prostate Test report examples. Green box highlighting the prognostic model score and Blue box highlighting the predictive model result for ADT benefit

Figure 3 shows an example of the test report. Figure 3a displays an example of an NCCN unfavourable IR prostate cancer who is estimated to have a low prognostic risk for distant metastasis and low predictive benefit for the addition of ADT with radiotherapy using the ArteraAI Prostate Test. Figure 3b displays the other side of the spectrum with the ArteraAI Prostate Test estimating a high prognostic risk for distant metastasis and high predictive benefit for the addition of ADT with radiotherapy. Before the test result is available, the pre-test shared ADT decision result is captured. After the ArteraAI Prostate Test results are discussed between the clinician and patient, the post-test shared ADT decision result will be recorded. At a median follow-up of 5 years for the cohort, a data linkage with PCOR will be established to allow for assessment of quality of life and efficacy data (Table 2).

**Table 2:** Schedule of assessments (according to SPIRIT-AI extension)

	Enrolment / Pre-test^1^	Post-test^2^	5-year follow-up
1. Demographics	X		
2. Prostate cancer history	X		
3. Shared ADT decision recommendation as determined by primary clinician and participant	X	X	
4. EPIC-26 Quality of Life questionnaire	X^*3*^		X^*3*^
5. Disease Control			X^*3*^

^*1*^ *Enrolment / pre-test can happen over a period of 14 days*

^*2*^ *Post-test data collection may occur at initial consult if ArteraAI Prostate Test results are available*

^*3*^ *Data linkage through Prostate Cancer Outcomes Registry at median 5-year follow-up.*

### Statistical considerations

Given the novel nature of ASTuTE, it is not possible to estimate the event rate for the primary endpoint proportion change in shared decision making regarding use of ADT. If an incorrect estimate is used, there is a risk of underpowering the study. Therefore, an interim analysis will be performed at 200 participants. This will allow for early assessment into the rates of management change and help determine the final number of participants needed to adequately power the final analysis of the study. At this time, 800 participants are planned for enrolment, with final numbers to be determined at the time of the interim analysis.

The McNemar’s test will be used to analyse the primary endpoint. There is a rare chance that the number of pre- to post-test recommendations of Yes-No and No-Yes is equal. This could lead to the null hypothesis being retained when the ArteraAI Prostate test is actually outperforming standard of care. As such, the study is designed to be hypothesis-generating, rather than focusing on a specific hypothesis that would define the sample size. This trial of implementation which assesses rates of management change based on a biomarker test has been used in recent literature. The DCISionRT^®^ study, used this approach to assess a breast genomic biomarker impact on radiation therapy recommendations [24].

### Data analysis plan

Simple statistical analysis will be performed by calculating “rates of change” with appropriate confidence intervals for changes in pre- and post-testing treatment recommendations. Summary statistics will be used to present the treatment recommendation pre- and post- incorporation of test results and secondary analyses.

**Fig. 4:**
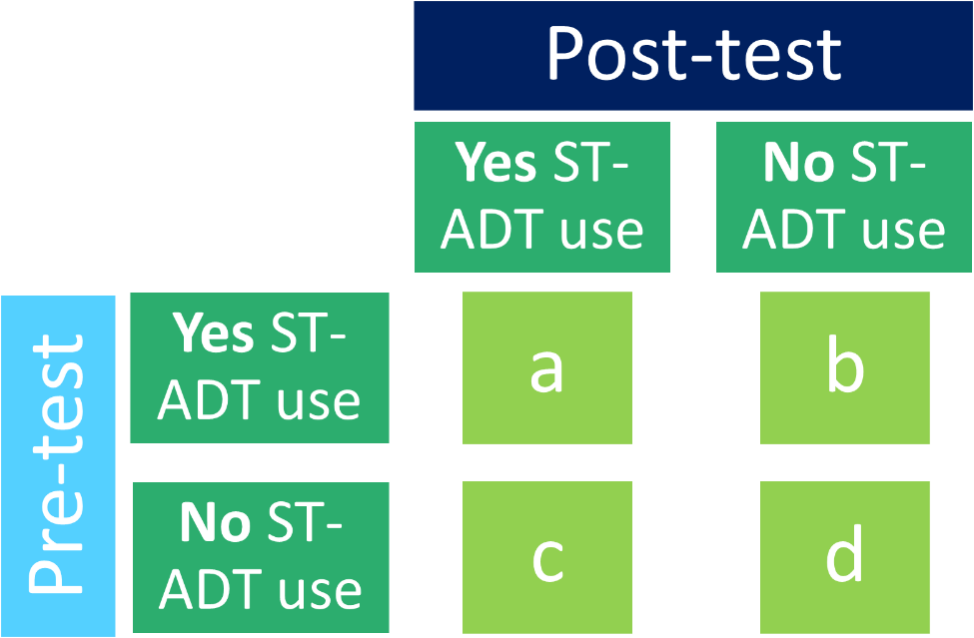
2 × 2 contingency table illustrating McNemar’s test for paired data to assess rates of change for the biomarker test, ArteraAI Prostate Test, on shared ADT decision making. Primary endpoint of proportion changing shared decision on use of ADT is calculated as (b + c) / (a + b + c + d)

For instance, in order to assess the impact of ArteraAI Prostate Test results on recommendations for shared ST-ADT use, the percentage change in recommendations will be calculated, and McNemar’s test for paired data will be used to assess the change in shared ST-ADT recommendations pre-test versus post-test, Fig. 4.

Multivariate logistic regression analyses will also be used to assess the odds ratios (OR) of factors leading to the pre-test and the post-test ADT recommendations. Pre-test exploratory covariates can include age, ISUP grade, initial PSA, tumour stage and percentage of cores positive. Post-test covariates will also include the ArteraAI Prostate test results.

The study opened to enrolment in December 2023, and is expected to complete accrual in 2025.

## Discussion

The question about the benefit of ST-ADT amongst IR prostate cancer patients undergoing radiotherapy is challenging due to the known toxicity profile of ADT, large range of potential disease outcomes for IR prostate cancer and unknown impact at an individual level of ST-ADT. IR prostate cancer is a large spectrum for staging in prostate cancers with varied outcomes amongst them [25]. MDACC and MSKCC retrospective data [26, 27] supports benefit for ST-ADT in only the unfavourable IR (UIR) men. However, some cases can be turned into UIR merely by changing the biopsy targeting method. At a population level, the most mature data from randomised control trials looking at the benefit for ST-ADT in IR men are summarised in Table 3. The limitation from these trials are the use of older radiotherapy techniques, heterogeneous patient populations, and the inability to identify and exact subpopulations that are the ones to benefit from the addition of ST-ADT.

**Table 3:** Pivotal ST-ADT randomised control trials for IR prostate cancer men

Trial Name	Number of patients, *n*	Intervention	Endpoints	Comments
**RTOG-9408** [3]	1086 IR men	66.6 Gy / 37 fractions ± 4 months of LHRH agonist with Flutamide	18 years bcF 57% woST-ADT 43% wST-ADT DSM 16% woST-ADT 9% wST-ADT	15 years PCSM: FIR vs. UIR 14% vs. 28% woST-ADT 9% vs. 12% wST-ADT DM: FIR vs. UIR 5% vs. 24% woST-ADT 8% vs. 10% wST-ADT
**EORTC 22,991** [10]	481 IR men	74 Gy / 37 fractions (71.1%) *or* 78 Gy / 39 fractions (28.9%) ± 6 months of LHRH agonist with bicalutamide for 7 days	12.2 years OS 74% woST-ADT 80% wST-ADT DM 27% woST-ADT 21% wST-ADT	
**RTOG 0815** [13]	1,492 IR men	79.2 Gy / 44 fractions (89%) *or* 45 Gy / 25 fractions with brachytherapy boost (11%) ± 6 months of LHRH agonist with antiandrogen for 10 days	8 years OS 79% woST-ADT 84% wST-ADT DM 4.3% woST-ADT 1.0% wST-ADT	− 67% having a single IR factor − 27% grade group 3 disease - excluded patients with more than two IR factors and ≥ 50% positive cores.
**DFCI 95–096** [11, 12]	153 IR men	70 Gy / 35 fractions ± 6 months of LHRH agonist with Flutamide	7 years OS with no or mild comorbidity 86% woST-ADT 91% wST-ADT OS with moderate or severe comorbidity 28.5% wST-ADT 62.5% wST-ADT	- Use of Adult Comorbidity Evaluation 27 comorbidity scores (ACE-27).
**PCS III** [28]	400 IR men	76 Gy / 38 fractions ± 6 months of LHRH agonist with bicalutamide	10 years PCSM 6.5% woST-ADT 1.5% wST-ADT DM 10% woST-ADT 3.5%% wST-ADT	-78% UIR -contained another randomisation arm of 70 Gy / 35 fractions wST-ADT, 200 IR men.

*Abbreviations*: IR - intermediate risk, LHRH - Luteinizing hormone–releasing hormone, bcF - biochemical failure, woST - ADT–without short–term androgen deprivation therapy, wST - ADT–with short–term androgen deprivation therapy, DSM - disease specific mortality, PCSM - prostate cancer specific mortality, FIR - favourable intermediate risk, UIR - unfavourable intermediate risk, DM - distant metastasis, OS - overall survival

Around 70% of men recover their testosterone from 6 months of ADT at 1.5 years from initial injection [29]. This is a lengthy time for reduced QoL and toxicity effects with some patients taking longer, or never recovering [30–33]. D’Amico et al. helped highlight the potential deleterious effects of ST-ADT in patients with moderate or severe comorbidity [11]. Although some of the effects on bone health and lean muscle mass can be proactively managed through serial DEXA imaging and exercise medicine, ideally only men most likely to derive meaningful benefit from ST-ADT would be exposed to these toxicities in the first place.

To better inform the clinician / patient discussion on ST-ADT better tools are required at the patient level to help personalise the anticipated benefit from ST-ADT. Implementation trials are becoming more necessary as technology evolves. DCISionRT^®^ was one of the first of these trials with the analysis of real world results on decision changes using a genomic biomarker. This trial was unique as it strongly showed how a validated biomarker can change clinical management decisions regarding the recommendation for adjuvant radiotherapy following breast conserving surgery in ductal carcinoma in situ (DCIS). Of the 539 women included 42% had changes to the adjuvant radiotherapy recommendation after this genomic biomarker test (46% yes to no, and 35% no to yes) [24]. Another implementation trial, GARUDA, characterised patients into low or high risk of developing late moderate to severe genitourinary toxicities following stereotactic body radiation therapy (SBRT) to the prostate by using a genomic biomarker test (PROSTOX). Of the 208 men included in this trial, 85% were classified as low risk and 15% as high risk. The vast majority of low risk patients, 98.8% chose SBRT, however, in the high risk cohort only 55.2% chose SBRT with the remainder choosing a moderately hypofractionated radiotherapy course (*p* < 0.001) [34].

A strategic objective in the ASTuTE trial was for the development of a robust platform where new technologies can be rolled out safely while assessing the degree of impact in a well-regulated environment. This opens the doors for a rapid adoption of machine learning, particularly in the context of digital histopathology. The ArteraAI prostate test has had the predictive model for ST-ADT validated [15] and the prognostic model validated [23]. It has outperformed the traditional NCCN classification, and is recommended as a tool in risk stratification with level 1B evidence. The ASTuTE trial will prospectively determine the utility of the ArteraAI Prostate Test in IR prostate cancer patients by measuring the rate of change in shared ST-ADT decisions and secondly will give more robust prospective data on efficacy whilst employing modern radiotherapy techniques. This is the first large scale deep learning technology to be employed to help guide prostate radiotherapy management and the success of this trial will enable other similar initiatives to follow suit

## Electronic supplementary material

Below is the link to the electronic supplementary material.

Supplementary Material 1

## Data Availability

No datasets were generated or analysed during the current study.
